# Isolation and characterization of filamentous fungi capable of degrading the mycotoxin patulin

**DOI:** 10.1002/mbo3.1373

**Published:** 2023-08-11

**Authors:** Megumi Mita, Rina Sato, Miho Kakinuma, Hiroyuki Nakagawa, Toshiki Furuya

**Affiliations:** ^1^ Department of Applied Biological Science, Faculty of Science and Technology Tokyo University of Science Chiba Japan; ^2^ Research Center for Advanced Analysis, Core Technology Research Headquarters National Agriculture and Food Research Organization Ibaraki Japan

**Keywords:** *Acremonium*, biocontrol, biodegradation, filamentous fungi, mycotoxin, patulin

## Abstract

Patulin is a toxic secondary metabolite synthesized by various fungal strains. This mycotoxin is generally toxic to microorganisms as well as mammals due to its reactivity with the important cellular antioxidant glutathione. In this study, we explored the presence of microorganisms capable of degrading patulin. Microorganisms were screened for the ability to both grow in culture medium containing patulin and reduce its concentration. Screening of 510 soil samples resulted in the isolation of two filamentous fungal strains, one of which, *Acremonium* sp. TUS‐MM1 was characterized in detail. Liquid chromatography‐mass spectrometry and nuclear magnetic resonance analyses revealed that TUS‐MM1 cells degraded patulin to desoxypatulinic acid. In addition, extracellular components of strain TUS‐MM1 also exhibited patulin‐transforming activity. High‐performance liquid chromatography analysis revealed that the extracellular components generated several products from patulin. Disc diffusion assay using *Escherichia coli* cells revealed that the patulin‐transformation products by the extracellular components are less toxic than patulin. We also demonstrated that a thermostable, low‐molecular‐weight compound within the extracellular components was responsible for the patulin‐transforming activity. These results suggest that strain TUS‐MM1 transforms patulin into less‐toxic molecules by secreting a highly reactive compound. In addition, once patulin enters the cells, strain TUS‐MM1 can transform it into desoxypatulinic acid to reduce its toxicity.

## Graphical abstract

Filamentous fungi capable of degrading patulin were isolated. *Acremonium* sp. TUS‐MM1 transforms patulin into less‐toxic molecules by secreting a highly reactive compound. In addition, once patulin enters the cells, strain TUS‐MM1 can transform it into desoxypatulinic acid to reduce its toxicity.

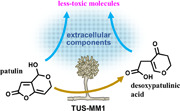

## 1 INTRODUCTION

Patulin is a toxic secondary metabolite synthesized by various fungi, including *Aspergillus, Byssochlamys*, and *Penicillium* strains. *Penicillium expansum* is the major producer of this mycotoxin in apples and other fruits. Patulin reportedly exhibits genotoxicity, immunotoxicity, and neurotoxicity in mammals (Pal et al., 2017; Saleh & Goktepe, 2019). Patulin contamination of foods and feeds is a serious worldwide problem. Therefore, many countries have established maximum tolerable levels of patulin contamination for fruit‐derived products (Vidal et al., 2019). For instance, the European Union set a maximum tolerable level of 50 μg/L in fruit juice and 10 μg/kg in products intended for consumption by children.

One mechanism of patulin toxicity involves reactivity with the important cellular antioxidant glutathione. The resulting depletion of glutathione leads to the accumulation of reactive oxygen species that damage cellular components (Ianiri et al., 2016). Thus, patulin is toxic to microorganisms as well as mammals. However, some microorganisms are reportedly resistant to patulin and occasionally degrade it to less‐toxic compounds. For instance, the basidiomycetous yeasts *Rhodosporidium kratochvilovae* and *Rhodosporidium paludigenum* degraded patulin to desoxypatulinic acid (Castoria et al., 2011; Pinedo et al., 2018; Zhu et al., 2015). In contrast, the ascomycete yeasts *Saccharomyces cerevisiae* and *Pichia caribbica* degrade patulin to ascladiol (Li et al., 2018; Zheng et al., 2018). Strains of the bacteria *Gluconobacter oxydans* and *Lactobacillus plantarum* also transform patulin to ascladiol (Hawar et al., 2013; Ricelli et al., 2007). Recent reports indicate that ascladiol is also generated from patulin degradation by two *Pseudomonas* strains (Ren et al., 2021; Xing et al., 2022).

With regard to filamentous fungi, only one *Byssochlamys nivea* strain has been reported to degrade patulin (Zhang et al., 2016). However, no degradation products have been identified. Elucidating the pathways by which microorganisms degrade patulin would be helpful not only for enhancing understanding of the degradation mechanism in nature but also for facilitating the application of these organisms in biocontrol efforts. For instance, *R. kratochvilovae* LS11, which degrades patulin to desoxypatulinic acid, has been employed to reduce patulin concentrations in stored apples (Castoria et al., 2005).

In this study, we attempted to discover novel patulin‐degrading microorganisms. We first selected microorganisms capable of growing in culture medium containing patulin. We then examined patulin degradation in the culture medium using high‐performance liquid chromatography (HPLC). Using this approach, we isolated two filamentous fungal strains from 510 soil samples. The ability of one of these strains to degrade patulin was examined in detail, and a major degradation product was identified.

## 2 MATERIALS AND METHODS

### 2.1 Chemicals and cultivation media

Patulin was purchased from Fujifilm Wako Chemicals. Bacto yeast extract was purchased from Thermo Fisher Scientific. All other chemicals were of analytical grade. KG medium contained (per liter) (NH_4_)_2_SO_4_ (3 g), KH_2_PO_4_ (1.4 g), Na_2_HPO_4_ (2.1 g), MgSO_4_·7H_2_O (0.2 g), FeCl_2_·5H_2_O (10.6 mg), CaCl_2_·2H_2_O (8 mg), ZnSO_4_·7H_2_O (4 mg), MnCl_2_·4H_2_O (2 mg), CuSO_4_·5H_2_O (0.02 mg), KI (0.2 mg), Na_2_MoO_4_·2H_2_O (0.2 mg), CoCl_2_·6H_2_O (0.2 mg), H_3_BO_3_ (0.4 mg), and NaCl (10 mg) (pH 7.2) (Furuya et al., 2011, 2019). Potato sucrose agar (PSA) medium contained (per liter) potato (200 g), sucrose (10 g), and Bacto agar (10 g) (pH 5.6 or 7.6). LB agar medium contained (per liter) Bacto tryptone (10 g), Bacto yeast extract (5 g), NaCl (10 g), and Bacto agar (10 g) (pH 7.0).

### 2.2 Isolation of microorganisms

In the first screening, we isolated microorganisms capable of growing in the presence of patulin. Each soil sample (ca. 50 mg) was suspended in saline (1 mL), and a portion of the resulting suspension (20 µL) was inoculated into a microtube containing 200 µL of KG medium supplemented with 3 mM patulin and 0.05 g/L yeast extract as carbon sources. Patulin was dissolved in dimethyl sulfoxide (300 mM), and 2 µL of this solution was added to the medium to suspend patulin. Samples were cultivated at 30°C with shaking (10,000 rpm) for 7 days. Aliquots of turbid cultures were then transferred into fresh medium. After sub‐cultivation, single colonies were isolated from turbid cultures by spreading appropriately diluted culture samples onto PSA medium plates.

In the second screening, isolated microorganisms were screened for the ability to degrade patulin. Microorganisms were cultivated in KG medium supplemented with 3 mM patulin and 0.05 g/L yeast extract, as described above. After cultivation, patulin degradation was analyzed using HPLC, as described in Section 2.5 below. Strain TUS‐MM1 was selected in the first and the second screenings and deposited in the NITE Biological Resource Center (Chiba, Japan) as NBRC 115867. The isolated strains, TUS‐MM1 and TUS‐MM2, were taxonomically identified based on sequencing of the ITS1 region, as described previously (Ishida & Furuya, 2021; Kurokawa et al., 2021).

### 2.3 Preparation of cells and extracellular components

**Figure A1:**
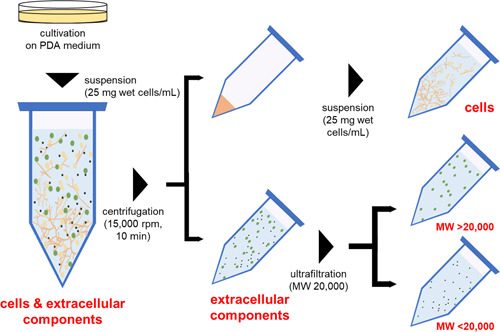
Preparation of cells and extracellular components of strain TUS‐MM1.

Strain TUS‐MM1 was cultivated on PSA medium at 30°C for 5 days, after which the cells were suspended in distilled water at a concentration of 25 mg wet cell weight/mL. This suspension consisted of cells and extracellular components and was divided into cell and extracellular component fractions by centrifugation (15,000 rpm, 10 min, 15°C) (Figure A1). The supernatant was collected, filtered through a 0.20‐µm pore size cellulose acetate membrane filter (Advantec Toyo, Tokyo, Japan), and used as extracellular components. The remaining cells were washed with distilled water and suspended in distilled water at a concentration of 25 mg wet cell weight/mL. This suspension consisted of only cells. The extracellular components were further ultra‐filtered using a 20,000 molecular weight (MW) membrane (Centricut W‐20, Kurabo, Osaka, Japan) and divided into retentate (MW > 20,000) and penetrate (MW < 20,000) fractions (Figure A1).

**Figure 1:**
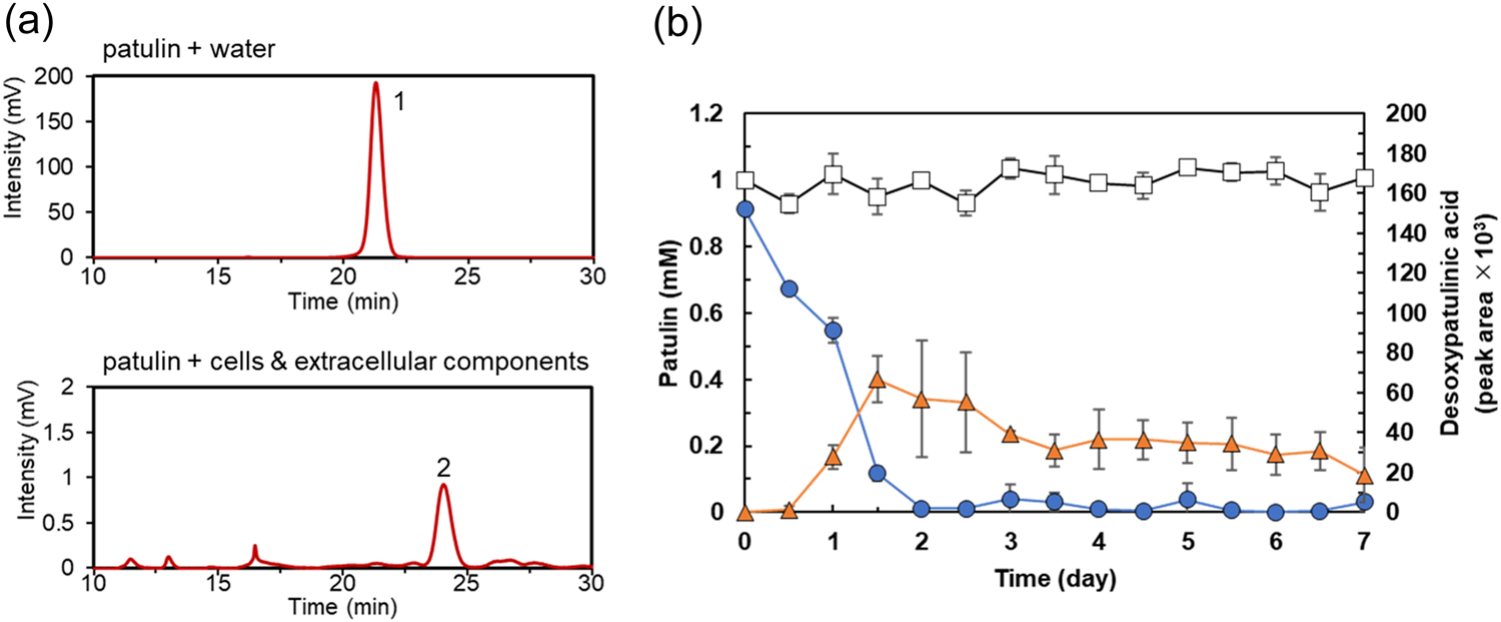
Analysis of reactions of the suspension containing both cells and extracellular components with patulin. (a) HPLC analysis. Water (upper panel) or suspension containing both cells and extracellular components (lower panel) was incubated with 1 mM patulin for 7 days. Peaks 1 (at 21 min) and 2 (at 23 min) corresponded to patulin and desoxypatulinic acid, respectively. (b) Time course of patulin transformation. The concentrations of patulin (circles) and desoxypatulinic acid (triangles) in the suspension containing both cells and extracellular components were determined at various time points. The concentration of patulin in water (squares) was also determined. Data are the average of three independent experiments, and error bars indicate the standard deviation of the mean. HPLC, high‐performance liquid chromatography.

### 2.4 Reactions using cells and extracellular components

Cells and/or extracellular components prepared as described above were used for reactions with patulin. The reaction mixture (200 μL) contained cells and/or extracellular components, patulin (1 mM), and dimethyl sulfoxide (DMSO) (1% [vol/vol]) in a test tube. The reactions were performed at 30°C with shaking (150 rpm).

### 2.5 Product analysis

Reaction products were analyzed by HPLC using an LC‐20 system (Shimadzu, Kyoto, Japan) equipped with a Cosmosil 5C18‐PAQ packed column (4.6 × 250 mm, Nacalai Tesque, Kyoto, Japan) (Kawana et al., 2022; Oya et al., 2022). The post‐reaction mixture (200 μL) was acidified by the addition of HCl (pH 2–3), and methanol (300 μL) was then added. The solution was vigorously shaken and centrifuged (15,000 rpm, 10 min, 15°C), and a portion (10 μL) of the resulting supernatant was injected into the HPLC system. The mobile phase was 0.005% formic acid aqueous solution, and the flow rate was 0.75 mL/min. Compounds were detected spectrophotometrically at a wavelength of 276 nm. The amount of patulin was calculated from a standard calibration curve prepared using a commercially available compound. In this process, patulin dissolved at various concentrations in DMSO was mixed in water. The amount of desoxypatulinic acid was expressed by peak area, since the purity of deoxypatulinic acid prepared as described below was not high enough (approximately 80%) to generate a standard calibration curve and further purification was unsuccessful.

The reaction product, desoxypatulinic acid, was purified using a Sep‐Pak Accell Plus QMA Plus Short Cartridge (360 mg sorbent per cartridge, 37–55 µm, Waters, Milford, MA, USA) according to the manufacturer's recommendations. The reaction product dissolved in methanol was applied to a cartridge equilibrated with methanol (5 mL). The cartridge was then washed with methanol (3 mL), and the bound reaction product was eluted with 2% acetic acid in methanol (3 mL). Liquid chromatography‐mass spectrometry (LC‐MS) analysis was performed using a JMS‐T100CS TOFMS system (JEOL, Tokyo, Japan) with electrospray ionization (Hashimoto et al., 2019; Ishida & Furuya, 2021). LC analysis was carried out as described above, with some modifications (flow rate, 0.2 mL/min; wavelength, 250 nm). Nuclear magnetic resonance (NMR) analysis was performed using a Bruker Spectrospin 400 system (Billerica, MA, USA), as described previously (Hashimoto et al., 2019; Nozawa et al., 2022).

Desoxypatulinic acid: ^1^H‐NMR (400 MHz, methanol‐*d*_4_): δ = 2.60 (t, J = 7.2 Hz, 2H, H‐3), 3.07 (d, J < 0.4 Hz, 2H, H‐7), 4.52 (t, J = 7.2 Hz, 2H, H‐2), 7.50 (t, J < 0.4 Hz, 1H, H‐6); ^13^C‐NMR (400 MHz, methanol‐*d*_4_): δ = 31.6 (C‐7), 36.9 (C‐3), 69.5 (C‐2), 114.1 (C‐5), 164.6 (C‐6), 175.7 (C‐8), 193.7 (C‐4); MS (ESI) (*m/z*): calculated for C_15_H_13_O_5_ [M‐H]^−^: 155.034; found: 155.035.

### 2.6 Disk diffusion assay

Patulin (0.5 mM) was incubated in water with or without extracellular components at 30°C with shaking (150 rpm) for 7 days. These solutions were subjected to disk diffusion assay to evaluate the toxicity of products generated from patulin transformation by the extracellular components. Cells of *Escherichia coli* JM109 were mixed with LB agar medium and poured into Petri plates. A volume of 30 µL of each solution prepared as described above was then impregnated into the paper disk with 6 mm diameter (Advantec Toyo), which was subsequently placed onto the LB‐agar plates. Water and ampicillin (5 mg/mL) were used as negative and positive controls, respectively. The plates were then incubated at 30°C for 2 days. The antibacterial activity was evaluated by measuring the diameters of growth inhibition zones.

## 3 RESULTS

### 3.1 Screening for patulin‐degrading microorganisms

Sub‐cultivation of microorganisms from 510 soil samples resulted in the isolation of 16 strains that grew in the presence of patulin. In the second screening, the isolated microorganisms were screened for the ability to degrade patulin. Two of the 16 isolated strains (TUS‐MM1 and TUS‐MM2) markedly reduced the amount of patulin in the culture medium.

Strains TUS‐MM1 and TUS‐MM2 were found to be filamentous fungi and taxonomically identified by sequencing of the ITS1 region. DNA fragments of ca. 250 bp were amplified from the genomic DNA of strains TUS‐MM1 and ‐MM2 using PCR. The ITS1 sequence of strain TUS‐MM1 exhibited 98% identity to those of *Acremonium pinkertoniae* CBS 157.70 (accession number NR_159611) and *Acremonium* sp. IFM 63143 (accession number LC317407). Based on these results, strain TUS‐MM1 was preliminarily identified as an *Acremonium* sp. The ITS1 sequence of strain TUS‐MM2 exhibited 99% identity to those of *Fusarium* sp. IFM 64894 (accession number LC512041) and *Fusarium* sp. IFM 65192 (accession number LC512043). Based on these results, strain TUS‐MM2 was preliminarily identified as a *Fusarium* sp. *Acremonium* sp. TUS‐MM1 exhibited high and stable patulin‐degrading activity and was thus chosen for further analyses.

### 3.2 Identification of a product generated from patulin degradation by TUS‐MM1

We analyzed the products generated by the incubation of patulin with *Acremonium* sp. strain TUS‐MM1. As strain TUS‐MM1 exhibited weak growth in liquid medium, cells cultivated on solid PSA medium were used for patulin‐degradation assays (Figure A1). TUS‐MM1 cells growing on PSA medium were suspended in distilled water. This suspension, consisting of both cells and extracellular components, was incubated with 1 mM patulin. HPLC analysis confirmed that patulin (retention time, 21 min) was completely consumed within 7 days (Figure 1a). Furthermore, post‐incubation HPLC analysis showed a new peak (retention time, 23 min) that was not detected in the control sample lacking strain TUS‐MM1 (Figure 1a). LC‐MS analysis revealed that the compound corresponding to this peak was a reduction (two‐hydrogen addition) product of patulin based on the determination of its mass ([M–H]^–^, *m/z* = 155.0). Furthermore, the ^1^H‐NMR and ^13^C‐NMR spectra of this product were in agreement with previously determined spectra of desoxypatulinic acid (Castoria et al., 2011). Based on these observations, the product generated from patulin degradation by strain TUS‐MM1 was identified as desoxypatulinic acid (see Figure 5).

Analysis of the time course of the patulin degradation (Figure 1b) revealed that the amount of desoxypatulinic acid increased as the amount of patulin decreased. Patulin was completely consumed within 2 days. The amount of desoxypatulinic acid produced from patulin gradually decreased from days 2 to 7.

### 3.3 Localization of patulin‐degrading activity in TUS‐MM1

**Figure 2:**
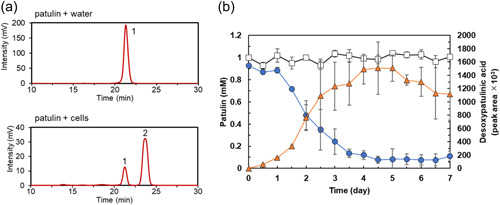
Analysis of reactions of the suspension containing cells with patulin. (a) HPLC analysis. Water (upper panel) or suspension containing cells (lower panel) was incubated with 1 mM patulin for 7 days. Peaks 1 (at 21 min) and 2 (at 23 min) corresponded to patulin and desoxypatulinic acid, respectively. (b) Time course of patulin transformation. The concentrations of patulin (circles) and desoxypatulinic acid (triangles) in the suspension containing cells were determined at various time points. The concentration of patulin in water (squares) was also determined. Data are the average of three independent experiments, and error bars indicate the standard deviation of the mean. HPLC, high‐performance liquid chromatography.

We investigated the localization of patulin‐degrading activity in strain TUS‐MM1. TUS‐MM1 cells growing in PSA medium were suspended in distilled water, and the suspension was divided into cell and extracellular component fractions by centrifugation (Figure A1). First, the cells were suspended in distilled water and incubated with 1 mM patulin. HPLC analysis confirmed that ca. 90% of the patulin (retention time, 21 min) was consumed within 7 days (Figure 2a). Furthermore, post‐incubation HPLC analysis showed a peak corresponding to desoxypatulinic acid (retention time, 23 min), as described in Section 3.2 above. These results demonstrate that TUS‐MM1 cells transform patulin into desoxypatulinic acid.

Analysis of the time course of the patulin degradation by TUS‐MM1 cells (Figure 2b) revealed that the amount of desoxypatulinic acid increased as the amount of patulin decreased. Almost all patulin (90%) was consumed within 5 days (Figure 2b), which was longer than the time required by the suspension containing both cells and extracellular components (2 days, Figure 1b).

**Figure 3:**
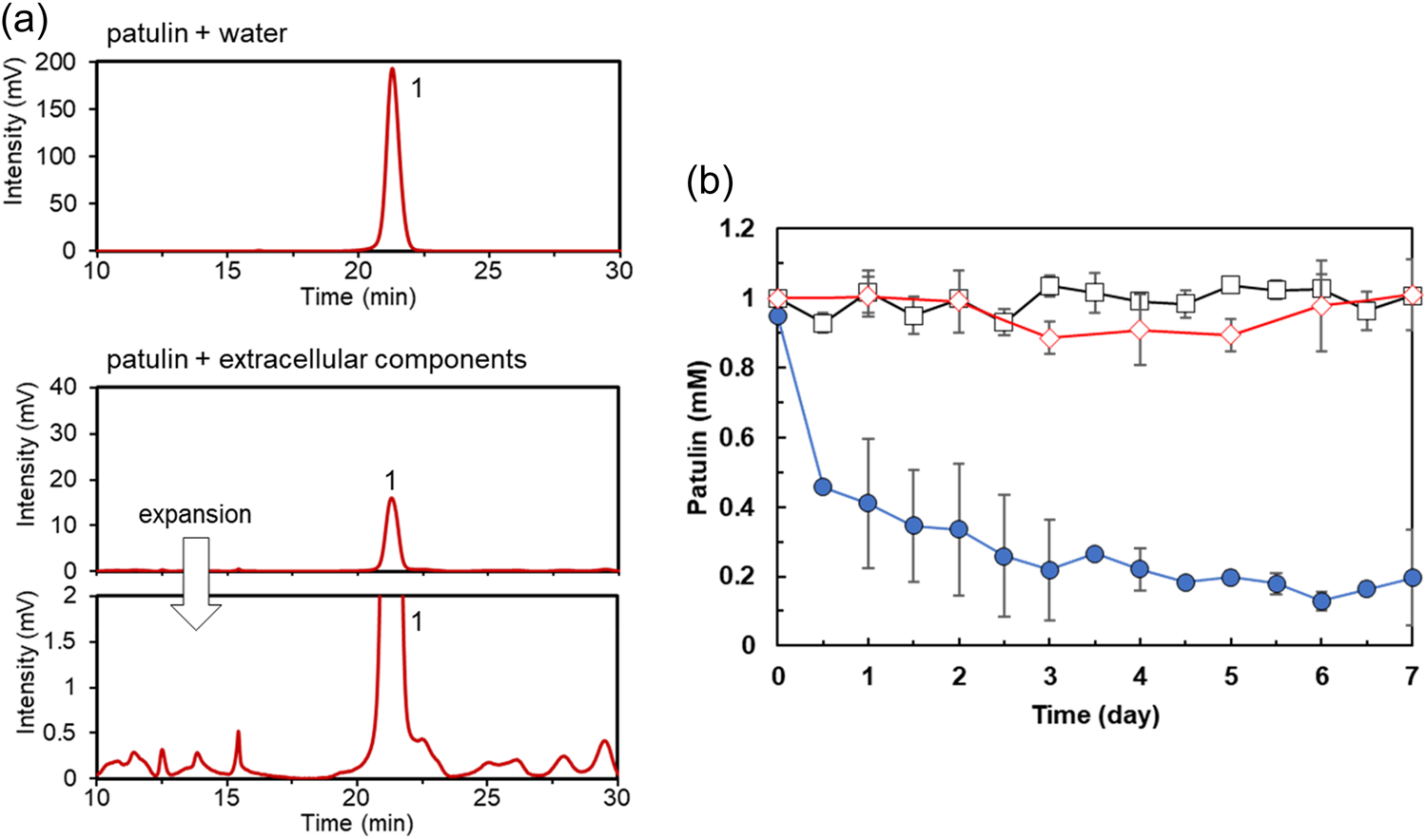
Analysis of reactions of the solution containing extracellular components with patulin. (a) HPLC analysis. Water (upper panel) or solution containing extracellular components (lower panel) was incubated with 1 mM patulin for 7 days. Peak 1 (at 21 min) corresponded to patulin. (b) Time course of patulin transformation. The concentration of patulin (circles) in the solution containing extracellular components (pH 7.2) was determined at various time points. The concentration of patulin in water at pH 5.9 (squares) and at pH 7.2 (diamonds) was also determined. Data are the average of three independent experiments, and error bars indicate the standard deviation of the mean. HPLC, high‐performance liquid chromatography.

We then examined the patulin‐transforming activity of the extracellular components of strain TUS‐MM1. The extracellular components were incubated with 1 mM patulin. Interestingly, HPLC analysis revealed that ca. 80% of the patulin was consumed within 7 days (Figure 3a). Furthermore, post‐incubation HPLC analysis showed several new small peaks corresponding to patulin‐transformation products. However, desoxypatulinic acid was not observed among these peaks. These results indicate that extracellular components of strain TUS‐MM1 transform patulin into several compounds.

Analysis of the time course of patulin transformation by the extracellular components of strain TUS‐MM1 (Figure 3b) revealed that 81% of the patulin was consumed in 3 days. The pH of the solution containing extracellular components of strain TUS‐MM1 was 7.2. We confirmed that patulin was not transformed in the absence of the extracellular components at pH 7.2 (Figure 3b). We also confirmed that patulin was not reduced in distilled water containing scrapings of PSA medium. These results demonstrate that not only TUS‐MM1 cells but also extracellular components secreted by the cells transform patulin.

**Figure A2:**
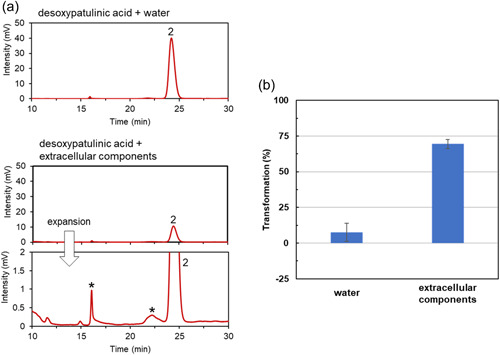
Analysis of reactions of the solution containing extracellular components with desoxypatulinic acid. (a) HPLC analysis. Water (upper panel) or solution containing extracellular components (lower panel) was incubated with desoxypatulinic acid for 7 days. Peak 2 (at 23 min) corresponded to desoxypatulinic acid. Peaks shown by an asterisk are detected also in the reaction mixture without extracellular components. (b) Transformation of desoxypatulinic acid. Water or solution containing extracellular components was incubated with desoxypatulinic acid for 7 days. Data are the average of two independent experiments, and error bars indicate the standard deviation of the mean. HPLC, high‐performance liquid chromatography.

We also examined the ability of the extracellular components to transform the major patulin‐degradation product, desoxypatulinic acid. When extracellular components were incubated with desoxypatulinic acid, 70% of the compound was transformed within 7 days (Figure A2).

### 3.4 Analysis of products generated from patulin transformation by the extracellular components of strain TUS‐MM1

**Figure A3:**
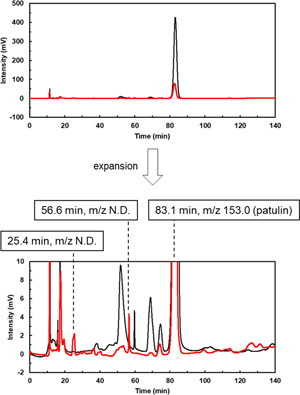
LC‐MS analysis of reactions of the solution containing extracellular components with patulin. After incubating 1 mM patulin with the extracellular components for 0 or 7 days, the solutions were concentrated 10 times and subjected to LC‐MS analysis. LC chromatograms (flow rate, 0.2 mL/min; wavelength, 250 nm) on days 0 and 7 are shown by black and red lines, respectively. N.D., the m/z value could not be determined due to low peak intensity.

We analyzed the products generated by the incubation of patulin with the extracellular components of strain TUS‐MM1. After incubating 1 mM patulin with the extracellular components for 7 days, the solution was concentrated 10‐fold and subjected to LC‐MS analysis. LC analysis of the reaction showed several peaks (Figure A3). Among them, 2 peaks (retention times of 25.4 and 56.6 min) were detected on day 7, but not on day 0, suggesting that these peaks correspond to products generated from patulin transformation. However, the m/z value could not be determined due to low peak intensity. Anyway, patulin is likely to be transformed into multiple compounds with small peaks by the extracellular components.

**Figure A4:**
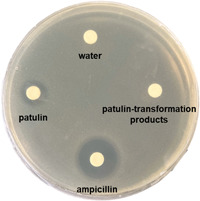
Analysis of toxicity of patulin‐transformation products by disk diffusion assay. The paper disks impregnated with the solutions (30 µL) containing 0.5 mM patulin or the patulin‐transformation products generated from 0.5 mM patulin by the extracellular components were placed onto an *E. coli* agar plate for an incubation period of 48 h. Water (30 µL) and ampicillin (5 mg/mL, 30 µL) were used as negative and positive controls, respectively. Diameters of the growth inhibition zone including paper disk (6 mm) were measured and described in Table 1.

The toxicity of products generated from patulin transformation by the extracellular components was evaluated by disc diffusion assay using *E. coli* cells. The paper disks impregnated with the solutions containing patulin or patulin‐transformation products were placed onto an *E. coli* agar plate for an incubation period of 48 h. Patulin inhibited the growth of *E. coli* (Table 1 and Figure A4), as previously reported (Wright et al., 2014). In contrast, the patulin‐transformation products did not affect the growth of *E. coli* (Table 1 and Figure A4). These results indicate that products generated from patulin transformation by the extracellular components are less toxic than patulin.

**Table 1:** Analysis of toxicity of patulin‐transformation products by disk diffusion assay.^a^

Sample	Diameter of growth inhibition zone (mm)
Patulin	12.3 ± 0.4
Patulin‐transformation products	0
Water	0
Ampicillin	17.0 ± 0.7

^a^ The paper disks impregnated with the solutions (30 µL) containing 0.5 mM patulin or the patulin‐transformation products generated from 0.5 mM patulin by the extracellular components were placed onto an *E. coli* agar plate for an incubation period of 48 h. Water (30 µL) and ampicillin (5 mg/mL, 30 µL) were used as negative and positive controls, respectively. Diameters of the growth inhibition zone including paper disk (6 mm) were measured. Data are the average of four independent experiments, and error bars indicate the standard deviation of the mean.

**Figure 4:**
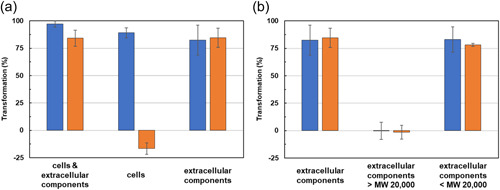
Analysis of thermal stability of patulin‐degrading activity of TUS‐MM1. (a) Cells and/or extracellular components were examined. (b) Fractions after ultrafiltration (20,000 MW membrane) of extracellular components were examined. The solution before (blue bars) or after (orange bars) autoclaving was incubated with 1 mM patulin for 7 days. In (a), negative degradation for autoclaved cells means that patulin in solution was slightly concentrated due to volume reduction associated with the removal of cells during HPLC sample preparation. Data are the average of three independent experiments, and error bars indicate the standard deviation of the mean. HPLC, high‐performance liquid chromatography; MW, molecular weight.

### 3.5 Thermal stability of patulin‐degrading activity of TUS‐MM1

We also evaluated the thermal stability of the patulin‐degrading activity of strain TUS‐MM1. A suspension containing both cells and extracellular components was autoclaved and then incubated with 1 mM patulin for 7 days. Surprisingly, autoclaving had almost no effect on the patulin‐degrading activity of the suspension (Figure 4a). We then examined the effect of autoclaving on the patulin‐degrading activity of TUS‐MM1 cells in the absence of extracellular components. Patulin was not consumed by autoclaved cells (Figure 4a). These results indicate that the patulin‐degrading activity of TUS‐MM1 cells is heat labile. We also examined the effect of autoclaving on the patulin‐transforming activity of the extracellular components. Autoclaving did not affect the patulin‐transforming activity of the extracellular components. These results demonstrate that the patulin‐transforming activity of the extracellular components is heat stable.

We also determined the general molecular weight of the compound within the TUS‐MM1 extracellular components responsible for patulin transformation using ultrafiltration. The extracellular components were divided into retentate and penetrate fractions using a 20,000 MW membrane filter (Figure A1). The patulin‐transforming activity was contained in the penetrate and exhibited high thermal stability (Figure 4b). These results indicate that a compound with MW of <20,000 is responsible for patulin transformation outside TUS‐MM1 cells.

## 4 DISCUSSION

In this report, we described the isolation and characterization of microorganisms that degrade patulin. The screening procedure resulted in the isolation of strains TUS‐MM1 and ‐MM2. It is interesting to note that both of the isolated strains were filamentous fungi. These strains were able to grow in the presence of a high concentration (3 mM) of patulin in liquid mineral medium supplemented with a low concentration (0.05 g/L) of yeast extract. We could not determine whether strains TUS‐MM1 and ‐MM2 utilize patulin as a carbon source, because the strains showed weak growth in liquid KG medium, irrespective of the presence of patulin and yeast extract. More‐detailed characterizations, including isotopic labeling studies, are needed to clarify whether these strains utilize patulin as a carbon source.

Strains TUS‐MM1 and ‐MM2 were identified as members of the genera *Acremonium* and *Fusarium*, respectively. Both of these filamentous fungi are classified within the order *Hypocreales*. To date, only one filamentous fungal strain, *B. nivea* FF1‐2, of order *Eurotiales*, has been reported to degrade patulin (Zhang et al., 2016). However, before the present study, no degradation products had been identified. In this study, we characterized strain TUS‐MM1 in detail to identify the degradation products of patulin and the components of this strain responsible for patulin degradation.

We found that *Acremonium* sp. TUS‐MM1 cells degraded patulin to desoxypatulinic acid (Figures 1 and 2). With regard to eukaryotes, it was reported that the basidiomycetous yeasts *R. kratochvilovae* and *R. paludigenum* degrade patulin to desoxypatulinic acid (Castoria et al., 2011; Pinedo et al., 2018; Zhu et al., 2015), and that the ascomycete yeasts *S. cerevisiae* and *P. caribbica* reportedly degrade patulin to ascladiol (Li et al., 2018; Zheng et al., 2018). These results suggest that the patulin‐degradation mechanism of the filamentous fungus *Acremonium* sp. TUS‐MM1 is similar to that of the basidiomycetous yeasts. Desoxypatulinic acid was confirmed to be less toxic than patulin in studies using human cells and *Escherichia coli* cells (Castoria et al., 2011; Wright et al., 2014). Castoria et al. (2011) also demonstrated that desoxypatulinic acid does not react with glutathione, in contrast to patulin. Thus, it is possible that strain TUS‐MM1 resists patulin by transforming this compound to desoxypatulinic acid.

Extracellular components of strain TUS‐MM1 also exhibited patulin‐transforming activity (Figure 3). HPLC analysis revealed that the extracellular components transformed patulin to generate several products. Interestingly, disc diffusion assay using *E. coli* cells revealed that the patulin‐transformation products by the extracellular components are less toxic than patulin (Table 1 and Figure A4). The amount of desoxypatulinic acid accumulated by the reaction of patulin with the suspension containing both cells and extracellular components (peak area ca. 60 × 10^3^, Figure 1b) was approximately 23 times lower than that accumulated by the reaction of patulin with only cells of strain TUS‐MM1 (peak area ca. 1400 × 10^3^, Figure 2b). Furthermore, we confirmed that desoxypatulinic acid was transformed by the extracellular components (Figure A2). These results indicate that extracellular components of strain TUS‐MM1 transform not only patulin but also desoxypatulinic acid. Based on the structural similarities of patulin and desoxypatulinic acid, these compounds might be transformed in a similar manner.

We demonstrated that a compound within the extracellular components responsible for patulin transformation was thermostable and of relatively low molecular weight (Figure 4). It was recently reported that the low‐molecular‐weight compound hydrazine produced by a *Pseudomonas* strain plays a crucial role in the degradation of another mycotoxin aflatoxin (Yao et al., 2021). Because attempts to identify the compound responsible for patulin transformation by strain TUS‐MM1 were unsuccessful due to low yield, further examinations of cultivation conditions and purification methods are needed to increase the yield of this compound.

**Figure 5:**
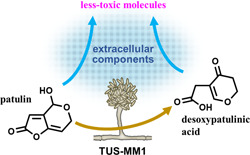
Proposed way of patulin degradation by TUS‐MM1.

In conclusion, we isolated filamentous fungi capable of degrading patulin. We demonstrated that not only cells but also extracellular components of *Acremonium* sp. TUS‐MM1 transform patulin (Figure 5). In other words, strain TUS‐MM1 transforms patulin into less‐toxic molecules by secreting a highly reactive compound. In addition, once patulin enters the cells, strain TUS‐MM1 can transform it into desoxypatulinic acid to reduce its toxicity. This strain is the first filamentous fungus shown to be capable of degrading patulin via desoxypatulinic acid. The degradation mechanism might be common and play an important role in mycotoxin detoxification by a variety of filamentous fungal strains. Furthermore, strain TUS‐MM1 and its components exhibit potential for application to the biocontrol of patulin contamination.

## AUTHOR CONTRIBUTIONS

**Megumi Mita**: Conceptualization (supporting); Data curation (lead); Formal analysis (lead); Investigation (equal); Methodology (equal); Resources (equal); Software (equal); Validation (equal); Visualization (equal); Writing – original draft (supporting); Writing – review & editing (equal). **Rina Sato**: Conceptualization (supporting); Data curation (lead); Formal analysis (lead); Investigation (equal); Methodology (equal); Resources (equal); Software (equal); Validation (equal); Visualization (equal); Writing – original draft (supporting); Writing – review & editing (equal). **Miho Kakinuma**: Conceptualization (supporting); Data curation (lead); Formal analysis (lead); Investigation (equal); Methodology (equal); Resources (equal); Software (equal); Validation (equal); Visualization (equal); Writing – original draft (supporting); Writing – review & editing (equal). **Hiroyuki Nakagawa**: Conceptualization (supporting); Investigation (equal); Methodology (equal); Validation (equal); Visualization (equal); Writing – original draft (supporting); Writing – review & editing (equal). **Toshiki Furuya**: Conceptualization (lead); Data curation (supporting); Formal analysis (supporting); Funding acquisition (lead); Investigation (equal); Methodology (equal); Project administration (lead); Resources (equal); Software (equal); Supervision (lead); Validation (equal); Visualization (equal); Writing – original draft (lead); Writing – review & editing (equal).

## CONFLICT OF INTEREST STATEMENT

None declared.

## ETHICS STATEMENT

None required.

## Data Availability

All data generated or analyzed during this study are included in this article. The nucleotide sequences of the ITS1 region of the isolated fungi, TUS‐MM1 and TUS‐MM2, are available in GenBank under accession numbers LC719793 and LC719794, respectively.

## 1

See Figure A1, Figure A2, Figure A3, Figure A4.
